# Flap Thickness and the Risk of Complications in Mechanical Microkeratome and Femtosecond Laser In Situ Keratomileusis: A Literature Review and Statistical Analysis

**DOI:** 10.3390/diagnostics11091588

**Published:** 2021-08-31

**Authors:** Piotr Kanclerz, Ramin Khoramnia

**Affiliations:** 1 Department of Ophthalmologyul, Hygeia Clinic, Jaśkowa Dolina 57, 80-286 Gdańsk, Poland; 2 The David J. Apple International Laboratory for Ocular Pathology, Department of Ophthalmology, University of Heidelberg, 69120 Heidelberg, Germany; Ramin.Khoramnia@med.uni-heidelberg.de

**Keywords:** femtosecond laser, laser in situ keratomileusis, complications, flap thickness, mechanical microkeratome

## Abstract

Introduction: A recent Cochrane review found no difference in visual acuity outcomes between femtosecond-assisted laser in situ keratomileusis (LASIK) and LASIK using mechanical microkeratomes (MMKs). This study compares the flap thickness and risk of complications related to flap creation using femtosecond lasers and MMKs. Methods: PubMed and the Web of Science are used to search the medical literature. An extensive search is performed to identify the flap thickness and complications of LASIK as reported up to 15 July 2021. The following keywords are used in various combinations: Corneal flap, femtosecond laser, laser in situ keratomileusis, laser-assisted in situ keratomileusis, LASIK, mechanical microkeratome. Results: After removing duplicates and irrelevant studies, 122 articles were included for review. Pooled differences for intended vs. postoperative flap thickness using MMKs and femtosecond laser were −4.07 μm (95% CI: −19.55, 3.24 μm) in studies on the MMK and 5.43 μm (95% CI: 2.30, 7.84 μm; *p* < 0.001), respectively. After removing the studies evaluating outcomes of the old generation Hansatome MMKs (which had a significantly greater variation of flap thickness), the pooled difference for newer MMKs was 4.97 μm (95% CI: 0.35, 9.58 μm; *p* < 0.001), but the results still favored the femtosecond laser. Uncommon and mild complications unique for the femtosecond LASIK are epithelial gas breakthrough, opaque bubble layer, transient light sensitivity syndrome, and rainbow glare. A single study reported a very low, but stastically different risk of postoperative flap slippage (0.033% for MMK LASIK, and 0.003% for femtosecond LASIK, respectively). Conclusion: In both manual microkeratome and femtosecond LASIK, intra- and postoperative complications were uncommon. The evidence of the superiority of one technique in terms of complications over another cannot be indisputably stated.

## 1. Introduction

Since introducing excimer lasers for refractive surgery, several million people have been successfully treated to decrease or eliminate their dependency on glasses or contact lenses to correct their ametropia [1]. Surface ablation procedures, such as photorefractive keratectomy (PRK) or laser epithelial keratomileusis (LASEK) and epi-LASIK, can be used very effectively to correct ametropia [2], and the risk for the development of keratectasia in uncompromised corneas is low [3]. However, patients’ visual acuity recovery is rather slow, and patients suffer from quite severe pain in the postoperative period. Therefore, laser-assisted in situ keratomileusis (LASIK) has become one of the most popular corneal refractive surgeries. It has proven to be a very safe and precise surgical technique offering rapid visual recovery, minimal postoperative pain, low risk for corneal haze, and reliable results for the correction of ametropia [4]. In LASIK, a flap is created with a femtosecond laser or mechanical microkeratome and then lifted, after which excimer laser energy is applied to the deeper corneal stroma [5]. Accuracy in corneal flap cutting is crucial for successful LASIK surgery.

Mechanical microkeratomes (MMKs) employ high-precision blade systems to create a lamellar corneal flap while the cornea is held under high pressure by a suction ring. MMKs have a long history of use in corneal refractive surgery and excellent flap-creation outcomes. MMKs employ translational motion systems to create nasal hinges and rotational systems to create a superior hinge. Moreover, the MMK plate can have an applanating effect (compressing the corneal tissues laterally) or indent the cornea (using a spherical molding system) for stabilization prior to cutting [6]. Disposable systems have a single-use head (housing the blade), and in some cases, disposable suction rings.

Femtosecond lasers operate at a wavelength of 1053 nm and apply infrared light pulses in a femtosecond (10^−15^ s) duration range on the tissue. The stromal layers are divided by photodisruption of the corneal tissue, which is a non-thermal process. The laser vaporizes small volumes of tissue, producing a shock wave, plasma, and a cavitation gas bubble that mainly consists of carbon dioxide, nitrogen, and water [7,8,9]. The first ophthalmic application of femtosecond lasers was in corneal surgery, with their subsequent adoption for cataract surgery as well [10]. Femtosecond lasers were considered revolutionary for corneal refractive surgery, and early results demonstrated great precision with low and adjustable corneal flap thickness. Because femtosecond lasers work by producing photodisruption of optically transparent tissue, such as the cornea [11], they can also be used for intrastromal treatment [12,13,14].

A recent Cochrane review found no difference in visual acuity outcomes between femtosecond-assisted laser in situ keratomileusis (femtoLASIK) and LASIK using mechanical microkeratomes [15]. Flap-related complications comprise the majority of complications in LASIK both intraoperatively, and postoperatively [16]. This study compares the flap thickness and risk of complications related to flap creation using femtosecond lasers and MMKs.

## 2. Methods

### 2.1. Literature Search

PubMed and the Web of Science were the resources used to search the medical literature. An extensive search was performed to identify the flap thickness and complications of LASIK in clinical and laboratory studies as reported up to 15 July 2021; limited searches were done after this date. The following keywords were used in various combinations: Corneal flap, femtosecond laser, laser in situ keratomileusis, laser-assisted in situ keratomileusis, LASIK, mechanical microkeratome. Only articles having English-language abstracts were included. The reference lists of identified publications were also considered potential sources of relevant articles. Studies were critically reviewed to create an overview and guidance for further search. No attempts to discover unpublished data were made. In addition to the search, selected chapters from relevant textbooks and trials registered in the ClinicalTrials.gov were included, if necessary. Emphasis was placed on articles published, since the review by Chen et al. [17], Farjo et al. [18], and Stonecipher et al. [19]; however, we also included earlier published articles to provide a comprehensive image of the results.

### 2.2. Study Selection for the Statistical Analysis

Articles were included in our statistical analysis if they met the following criteria: (i) The study analyzed postoperative flap thickness in MMK or femto LASIK; (ii) the study presented the intended flap thickness with the evaluated method; (iii) the true flap thickness was measured up to 6 months postoperatively; and (iv) the difference between the measured and the intended flap thickness was reported (with a corresponding 95% confidence interval [CI], or the study provided data to calculate them). If an identified article reported outcomes using more than two methods (e.g., with two different MMK devices, or with different flap-thickness planned with the femtosecond laser) the different methods were presented within Table 1, and the results for each method were plotted in the figures. If an investigation analyzed measurements at different timepoints, usually the last measurement was taken into account. The names of the devices are stated as reported in the articles, with no intent to modify it. As only one study was a randomized controlled trial [20], a classical meta-analysis was not possible, so we included randomized, non-randomized, and retrospective studies. Our statistical analysis investigated effect sizes (i.e., differences between intended and postoperative flap thickness) using a DerSimonian-Laird random-effects model. Heterogeneity was assessed using the I2 test. High heterogeneity among the individual study results necessitated the application of DerSimonian-Laird random-effects modelling. Pooled differences between the two surgical techniques were tested by the chi-squared test. A level of *p* < 0.05 was considered statistically significant. Because the risk of complications in some studies was approximately 1%, we estimated that studies having a sample size of at least 381 eyes would be required to determine real values within ±1%. Therefore, within the tables analyzing the risk of intraoperative and postoperative complications of LASIK we have analyzed only studies with a minimum of 381 eyes. With that, if a study did not present an overall risk of complications, but only a rate of complications within specified periods e.g., to assess a trend for analyzing the change over time [16,21,22], it was not included in the tables.

## 3. Results

The initial search identified 495 unique articles (Supplementary Materials). After removing duplicates and irrelevant studies, 120 articles were included for review.

### 3.1. Flap Thickness and Morphology in Manual Keratomes and Femtosecond Lasers

Flap thickness is a critical indicator for LASIK safety because of the importance of stromal preservation. Creating thinner and more predictable flaps extends the margin of safety in refractive surgery [23]; it maintains postoperative corneal strength and minimizes the risk of corneal ectasia [24]. Moreover, thinner flaps in myopic LASIK are associated with faster visual recovery and less myopic refractive outcome [25]. Only one study comparing flap thickness between MMK and a femtosecond laser was a randomized controlled trial; in an investigation of eyes in 21 patients undergoing bilateral keratomileusis a flap was created with MMK in one eye, and with the femtosecond laser in the other eye [26]. The flap thickness of the flap created with the Hansatome was smaller than intended, while the flap created with the IntraLase was thicker than planned with no statistical difference in variances [26].

**Figure 1:**
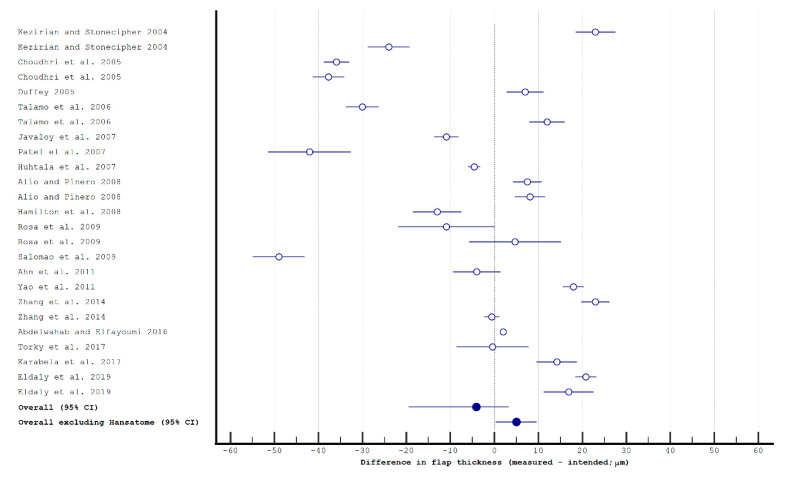
The flap thickness in mechanical microkeratome laser in situ keratomileusis.

**Figure 2:**
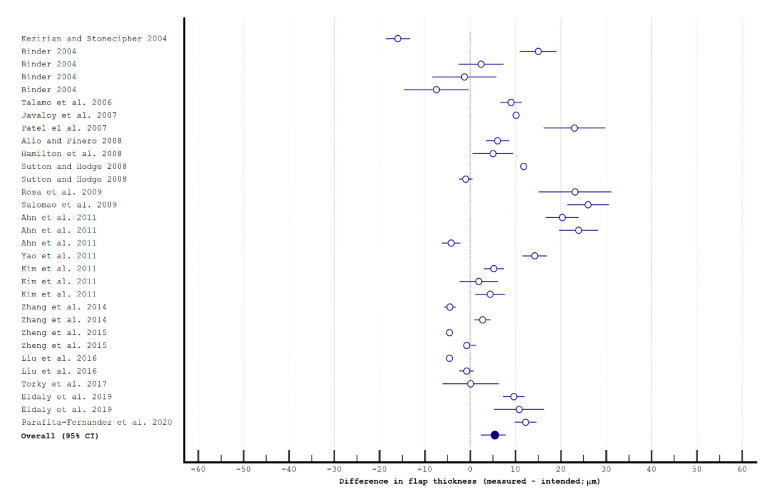
The flap thickness in femtosecond laser in situ keratomileusis.

The studies analyzing postoperative flap thickness using MMK and femtosecond LASIK are presented in Table A1. Pooled differences for intended vs. postoperative flap thickness were −4.07 μm (95% CI: −19.55, 3.24 μm) in studies about the MMK (Figure 1) and 5.43 μm (95% CI: 2.30, 7.84 μm; *p* < 0.001) and in studies about the femtosecond laser (Figure 2), respectively. The data heterogeneity (*I*^2^) of the studies was 99.15% and 99.47%, respectively. After removing the results for Hansatome MMKs, the pooled difference for manual MMKs was 4.97 μm (95% CI: 0.35, 9.58 μm; *I*^2^ = 98.55%); it was still significantly different from femtosecond lasers (*p* < 0.001) and favored them.

**Figure 3:**
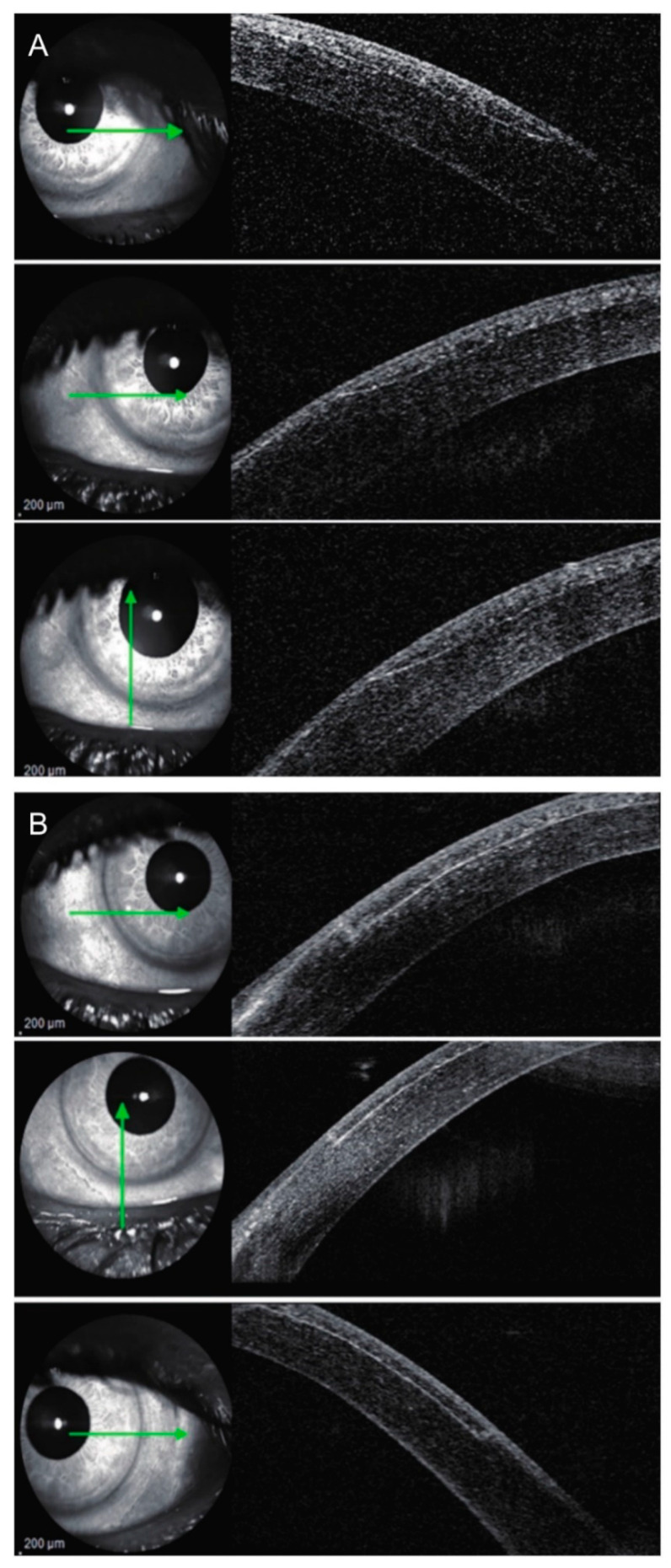
Laser in situ keratomileusis (LASIK) flap side-cut angle and edge outline in (**A**) microkeratome-assisted LASIK and (**B**) femtosecond laser-assisted LASIK groups by anterior segment optical coherence tomography (OCT). Green arrows indicate the location from where the OCT scans were obtained. Reproduced with permission from [28].

The configuration of flaps created using a femtosecond laser is different from those created with MMKs. For femtosecond-laser flaps, the central flap thickness is similar to the peripheral flap thickness; MMK flaps are meniscus-shaped, with the center of the flap thicker than the periphery [27]. This difference was reported in optical coherence tomography (OCT) studies that compared flaps created with the FS200 laser (Alcon, Fort Worth, TX, USA) and the M2 MMK (Moria, Doylestown, PA, USA) [28], as well as flaps created with the IntraLase (IntraLase, Irvine, CA, USA) and Zyoptix XP MMK (Bausch + Lomb, Rochester, NY) [29]. On the other hand, Ahn et al. noted that the femtosecond laser flap thickness was only minimally different between the center and the periphery for both the IntraLase and the VisuMax lasers (Carl Zeiss Meditec, Jena, Germany), but not for the Femto LDV (Ziemer Ophthalmic Systems, Port, Switzerland) laser, which also created meniscus-shaped flaps [27]. The peripheries of the flaps also differ (Figure 3); with MMKs the periphery has an oblique configuration, while with the femtosecond laser, it is perpendicular to the corneal surface [28]. Corneal biomechanics, expressed by corneal hysteresis and a corneal resistance factor, change similarly after femtoLASIK and MMK-LASIK treatment [30]. Nevertheless, flap creation using a femtosecond laser caused more predictable corneal biomechanical changes than using an MMK [30]. 

The literature review indicates that the femtosecond laser has a tendency to create a slightly thicker flap than intended, while with MMKs the flap is thinner than intended. Some authors have suggested that this may partially be due to the use of the 180 µm head of the Hansatome, which could tend to create a larger than intended flap thickness [26,31,32]. Nevertheless, excluding studies evaluating outcomes of the Hansatome MMK did not significantly change the results. Not only femtosecond lasers, but also microkeratomes have been improved over the last years; current MMKs can create thinner and more predictable flaps with lower variations in flap thickness [23]. For example, in a study by Zhang et al. that included a newer Moria One-Use Plus MMK, the difference between MMK and femtosecond flap thickness was 2.6 ± 9.1 μm, which was not statistically significant (*p* = 0.12) [33]. Moreover, the standard deviations for flap thickness were similar between the MMK and femtosecond groups (6.8 μm vs. 7.2 μm, respectively). In the same study, only in the MMK group, flap thickness was linearly correlated with the central corneal thickness [33]. In a study by Huhtala et al., the flap thickness of flaps created with the Moria M2 MMK was moderately correlated with the preoperative corneal thickness (*r* = 0.536) [34]. In another investigation, the thickness of a flap created by a Moria LSK−1 MMK was weakly correlated with the central corneal thickness (R^2^ = 0.21), but no correlation was found for the Moria M2 or the IntraLase devices [35]. 

Another issue is the method and timing of flap thickness measurement. Rosa et al. have suggested waiting 20 min after femtosecond laser flap creation to achieve more reliable measurements of flap thickness by eliminating the influence of corneal dehydration [20]. Nevertheless, in most of the analyzed studies, flap thickness was calculated by subtraction pachymetry, i.e., confronting preoperative central corneal thickness with corneal thickness after flap lift. In some investigations, OCT [24,27,28,29,36,37,38,39] or confocal microscopy [26,40] was performed postoperatively to evaluate flap thickness. Although subtraction pachymetry could be considered as the gold standard for flap thickness evaluation [41], OCT measurements were found to be more accurate with a tighter standard deviation [42]. Potentially, ultrasound pachymetry is subject to edema and compression, which are not present in postoperative OCT measurements [42]. Studies reporting the change in flap thickness over time in the postoperative period showed ambiguous results. In the investigation by Yao et al. evaluating mainly myopic corrections, flap thickness at one week and one month postoperatively were greater than six months after surgery (114.24 ± 6.93 µm, 115.82 ± 11.21 µm and 100.16 ± 0.87 µm for femtosecond and 127.97 ± 7.57 µm, 126.42 ± 11.25 µm, and 112.18 ± 5.39 µm for MMK flaps, respectively) [37]. Another study found that the thickness of flaps created with the Alcon FS200 showed a trend for thickening over time (mean values 128.1 µm, 130.5 µm, and 132.2 µm, at 1 day, 1 month, and 3 months after surgery, respectively) [39]. Javaloy et al. noted minor changes in flap thickness from 1 to 3 months postoperatively (129.35 ± 3.43 to 130.14 ± 1.7 µm for the femtosecond laser, and 148.0 ± 16.74 to 149.08 ± 14.03 for the MMK, respectively) [40]. It is possible that a transient edema of the corneal tissue would increase the flap thickness in the immediate postoperative follow-up, and the edema would decrease over time, reducing flap thickness [39]. On the contrary, progressive thickening of the corneal epithelium centrally in the late postoperative period was reported after myopic LASIK. It was correlated with the level of corrected spherical refractive error [43]. For every diopter of spherical equivalent corrected, a 1.15 µm epithelial thickening was noted three months after LASIK [44]; this increase in epithelial thickness would potentially also affect the total flap thickness. Although it is believed that the femtosecond laser allows better centration of flaps compared to those created with MMKs, this hypothesis was not confirmed in our literature search. In a single study comparing the centration of flaps created with different femtosecond laser platforms, flaps created with the VisuMax laser were more nasally displaced compared to those created with Wavelight FS200 [45].

### 3.2. Risk of Intraoperative Complications Associated with Flap Creation

The rates for intraoperative, flap-related complications for both manual and femtosecond procedures are presented in Table 1; in some studies, it was related to the model of the device. For example, the risk for a buttonhole was significantly higher (*p* = 0.0001) for the Hansatome microkeratome (Bausch and Lomb Surgical., San Dimas, CA) than for the Automated Corneal Shaper (Chiron-Adatomed, Munich, Germany) [46], while another study found higher rates for the Hansatome MMK compared to the Moria LSK2 keratome (0.3% and 0.1%, respectively; *p* = 0.04) [47]. Despite these differences, in both of these studies, the total complication rates did not differ between the devices [47]. In another investigation, the overall rate of complications was higher for the Automated Corneal Shaper than for the Hansatome for all analyzed complications [48]. Than et al. have reported that the rate of intraoperative complications was higher during the surgeon’s first 1000 eyes (1.3%) compared to their last 1000 eyes (0.4%; *p* = 0.0481) [46]. Similarly, Lim and Maloney reported the incidence rate for intraoperative complications was 6.0% in the first 100 eyes, 2.3% in the next 600 cases, and then 0.3% in the last 300 eyes [49].

Using MMKs, free caps, incomplete flaps, and buttonhole flaps are well-known complications associated with flap creation, with incidence rates reaching less than 1%. Assessed by preoperative keratometric power, eyes with flatter corneas had more free-caps complications and incomplete flaps than eyes with steeper corneas, and eyes with steeper corneas had more epithelial abrasions and thin/irregular flaps than eyes with flatter corneas [50]. On the other hand, Nakano et al. reported that the overall rate of intraoperative complications was greater for steeper corneas, and was greater using the Automated Corneal Shaper (1.26%) compared to the Hansatome (0.63%) and MK-2000 (0.63%) MMKs (*p* < 0.001 and *p* = 0.03, respectively) [51]. Less significant MMK complications included epithelial abrasions (0.93%) [50], and irregular stromal edge cuts at the side of the hinge (0.019–2.0%) [52,53]. Incomplete flaps, free caps, and buttonholes can also occur during femtosecond LASIK, but free caps and buttonholes are associated with intraoperative flap manipulation, rather than flap creation, as seen with MMKs [54]. The risk for incomplete flaps using femtosecond LASIK was reported by Haft et al. to be 0.06% [55], which is lower than that for the present MMK studies. During femtosecond LASIK, an incomplete flap can result from the suction loss. A suction loss usually occurs during the raster pattern (early or late), and before the side cut is initiated [56]. In these cases, an immediate lamellar recut usually can be performed, with excellent visual outcomes. An advantage of femtosecond laser flap creation is the possibility to adjust the flap thickness, so that the surgery can be continued. In cases of incomplete flaps during LASIK with MMKs, the procedure should be terminated, and the flap left for healing [57,58].

Complications limited to femtosecond laser use are epithelial gas breakthrough [18] and opaque bubble layer (OBL) formation [1,59]. A vertical gas breakthrough occurs between the dissection plane and the subepithelial space, resulting in an escape of gas bubbles into the subepithelial space [60]. A focal break in Bowman’s layer (e.g., during a previous MMK LASIK procedure or a corneal scar) could induce an epithelial gas breakthrough [61]. The rate of such complications has been reported to be 0.25% [55]. In such cases, the femtosecond laser cannot photodisrupt the corneal stroma in a small portion of the interface, or if there is resistance within the interface from scar tissue [62,63]. On the other hand, an advantage of the MMKs is the possibility to create a flap even in opaque tissues [18]. Although uncommon, vertical gas breakthrough can be associated with epithelial downgrowth, corneal scarring, and microstriae [61]. OBL represents an accumulation of gas bubbles at the stromal interface; these bubbles cannot escape, due to corneal compression and the high levels of the vacuum created by applanation, leading to transient interface opacity. Courtin et al. reported an incidence rate for OBL of 48%, but the average affected area was less than 10% of the flap area [59]. In another study, 56.4% of eyes developed OBL; 32.2% of eyes had a diffuse OBL pattern, while 24.4% of eyes had hard OBL patterns [64]. The thickness of the central cornea, the corneal resistance factor, and corneal hysteresis were all positively correlated with the OBL area [59]. Excessive OBL may interfere with flap creation, measurements of residual stromal-bed thickness, and the laser tracking system, thus delaying the procedure [65]. One might consider if excessive OBL should not be considered as a true complication, since it is managed by just waiting a while; in a study by Liu et al., OBL did not influence postoperative visual acuity, but led to a mild decrease in postoperative contrast sensitivity [65]. An infrequent complication during femtoLASIK is anterior chamber gas bubbles; its mechanism is uncertain, but direct posterior pressure induced by the laser shockwave might push the gas through loose stromal lamellae or the episclera [66]. This complication might hamper the eye tracker responsible for centering the ablation. Hence, options are to wait for the bubbles to resolve, or to finish the treatment without an eye tracker [67].

Another problem associated with the creation of a LASIK flap is the increase in IOP during suction. Although the mechanical alterations during flap creation are unlikely to induce retinal detachment [68], the risks associated with the IOP elevation should be considered in high-risk patients, particularly with preexisting optic nerve damage [69]. The IntraLase platform induces a lower IOP to increase during suction than the Moria MMK (119.33 ± 15.88 mm Hg vs. 160.52 ± 22.73 mm Hg, respectively), although the time needed for creating the flap was more than twice as long (92.85 ± 13.49 s vs. 36.42 ± 7.48 s, respectively) [70]. In newer platforms, suction times may be slightly shorter: Salomão et al. reported it was 56 s for the 30 kHz platform, and 40 s for the 60 kHz laser [15]. The introduction of high repetition rate femtosecond lasers has decreased the time needed for flap creation [1,8,71]. The surgeon-related factor might be considered higher in MMK surgeries, and the complications of femtosecond laser flap creation could be less severe and easier to manage.

One might conclude that the overall complication rate is low during keratomileusis, both during MMK and femtosecond laser flap creation, with several studies reporting complication rates below 1.0% [46,48,53,55].

**Table 1:** Large clinical studies that analyze the rate of intraoperative flap complications in manual and femtosecond flap creation. The total risk of complications is stated only if calculated within the study.

Study	Method for Flap Creation	Number of Eyes *	Free Cap	Incomplete Flap	ButtonHole	Thin	Thick	Irregular Flap	Suction Loss	Epithelial Gas BreakThrough	Other
**Stulting et al., 1999** [72]	manual (Chiron Automated Corneal Shaper)	1244 ** (M)			0.5%	0.1%	0.1%	0.1%			0.0%
**Lin and Malloney 1999** [49]	manual (Chiron Automated Corneal Shaper)	1019 (M)	1.0%	0.3%				0.9%			
**Tham and Moloney 2000** [46]	manual (Bausch and Lomb Hansatome or Automated Corneal Shaper)	3988 (N/A)	0.1%	0.2%	0.1%	0.2%					0.1%
**Jacobs and Taravella 2002** [48]	manual (Bausch and Lomb Hansatome or Automated Corneal Shaper)	84,711 (N/A)	0.0%	0.1%	0.1%	0.1%					0.0%
**Nakano et al., 2004** [51]	manual (Nidek MK-2000, Bausch and Lomb Hansatome and Automated Corneal Shaper)	34,182	0.08%	0.23%	0.13%						
**Carrillo et al., 2005** [53]	manual (Nidek MK-2000)	26,600	0.086%	0.049%	0.049%			0.019%			0.019%
**Albeda-Vallés et al., 2007** [50]	manual (Moria LSK-1)	34,099 (M/H)	1.67%	0.36%	0.11%	0.82%					0.93%
**Al-Mezaine et al., 2011** [47]	manual (Bausch and Lomb Hansatome and Moria LSK2)	4352 (M/H)	0.1%	0.6%	0.2%						0.1%
**Haft et al., 2009** [55]	femtosecond (AMO IntraLase 15 and 30 kHz)	4772 (N/A)		0.06%#				0.02%	0.06%#	0.25%	

* preoperative refractive error, if stated (M)—Myopia; (H)—Hyperopia; ** 182 LASIK enhancement procedures were included in the analysis (not possible to exclude this group from the analysis), # due to suction loss.

### 3.3. Risk of Postoperative Complications Associated with Flap Creation

Postoperative complication rates for manual and femtosecond laser procedures are presented in Table 2. Several studies have reported a higher incidence of diffuse lamellar keratitis (DLK) with the femtosecond laser than with an MMK [17,18,73,74,75]. Tomita reported that DLK incidence also varied between different femtosecond platforms; it was 8.17% for the Femto LDV, and 37.5% for the IntraLase FS60 [76]. In most cases, only mild-stage (I/II) DLK was reported. Although patients with DLK were less likely to achieve the best-corrected visual acuity 20/20 or better one day postoperatively, the condition was self-limiting with a good prognosis [77,78]. The risk of developing DLK has been reported to be associated with higher raster energy used during the procedure, larger flap diameter, higher side-cut energy, blood in the interface, and flap epithelial defects [78,79]. On the other hand, intraoperative irrigation of the stromal interface with a dexamethasone 0.1% suspension or prednisolone sodium phosphate 0.5% solution significantly dropped the rate of DLK [80].

Another uncommon complication associated with femtosecond-laser flap creation is transient light-sensitivity syndrome (TLSS) [19]. The incidence of TLSS has been reported to be 0.25–1.3% [55,81,82]. In the current systems, which use low energy and high pulse rate, the risk of TLSS might be significantly lower than in the very first femtosecond laser systems that used high energy and low pulse rate [83]. Patients with TLSS usually present 2–6 weeks after surgery with good visual acuity, minimal slit lamp findings, and extraordinary light sensitivity [81]. The etiology of this condition may be associated with increased keratocyte activation at the interface, and a reduction of laser energy-reduced its incidence [81]. For this condition, aggressive topical steroid therapy is recommended, and symptoms usually resolve within a week without affecting final visual acuity [84].

Flap stability is a key safety concern in LASIK, and early flap displacements are very strongly undesirable complications. Animal studies have shown that the femtosecond laser-produced greater corneal stromal inflammation compared to an MMK postoperatively, and stronger flap adhesion 1–3 months postoperatively [85,86]. In a clinical study by Clare et al. [87], postoperative flap displacement (within 48 h after surgery) in eyes with no trauma was more common using an MMK (0.033%) compared to femtosecond LASIK (0.003%). Hyperopia was the strongest predictor for flap displacement (odds ratio 19.29) [87]. This increased MMK risk for flap displacement may be associated with flap characteristics; increased MMK flap thickness resulted in heavier flaps that increased rotational forces about the horizontal axis [87]. Femtosecond LASIK hinge placement (i.e., superior) has a potential role in stabilizing the flap, protecting the flap against slippage, in contrast to nasal hinge placement where folds potentially tend to occur at the hinge [88]. On the other hand, tear break-up time and Schirmer test results six months after LASIK were shown to be more severely aggravated by superior than nasal hinges [89]. Management of flap dislocation can range from light stroking with a moist surgical sponge or swelling the flap with hypotonic solutions to lifting, repositioning, or even suturing the flap [79].

Salamão et al. reported a higher incidence of dry eye syndrome following MMK keratomileusis compared to femtosecond LASIK (46% vs. 8%; respectively, *p* < 0.0001) in patients without preoperative dry-eye signs [31]. Although these patients were followed for nine months after surgery, the presence of corneal punctate epithelial erosions was only evaluated for one month after surgery [31]. In another study of 274 eyes, Schirmer test averages and tear break-up time averages did not differ between MMK and femtosecond laser flap-creations [90]. Neurotrophic effects from interrupting corneal innervation were considered important causative factors, as no correlation was found between flap thickness (or ablation depth) and the incidence of LASIK-induced dry eye in their study [31]. Moreover, Tanna et al. reported faster recovery (the percentage of eyes with 20/20, or higher uncorrected visual acuity, one day, one week, one month, and three months after surgery) after IntraLase FS 60 Hz treatment compared to keratomileusis with the Moria One Use-Plus MMK [91].

Another uncommon postoperative complication unique to the femtosecond laser is rainbow glare (RG), with a reported incidence of 5.8% [18,92,93]. RG is an optical phenomenon where the patient sees an array of spectral bands under mesopic and scotopic conditions following femtosecond LASIK. It is believed to be a consequence of a transmissive diffraction grating formed on the posterior surface of the corneal flap [92]. RG has occurred despite the fact that femtosecond lasers in general (but specifically the IntraLase FS150 and Wavelight FS200 in their study) created greater flap smoothness than the Carriazo-Pendular MMK assessed by scanning electron microscopy [94]. Greater flap smoothness is observed in newer, high-frequency lasers as they provide tighter spot/line separation [95,96]; despite these advances enabling narrower grating size, RG has also been reported in newer-generation lasers [93,97,98]. RG was reported to be associated with higher raster energy settings and time from the service maintenance visit (as it these visits focus the optics and ensure beam alignment). RG is a transient experience, and symptoms usually resolve by the ninth postoperative month [92]. For unresolved RG, lifting the flap and performing photorefractive keratectomy of the underlying surface should be considered along with just irrigating the interface [99,100].

**Table 2:** Large clinical studies that analyze the rate of postoperative complications in manual and femtosecond laser flap creation. The total risk of complications is stated only if calculated within the study.

Study	Method for Flap Creation	Number of Eyes *	Flap Displacement	Epithelial Ingrowth	Local Keratitis (Culture Positive or Negative)	Flap Folds	DLK	TLSS
**Stulting et al., 1999** [72]	manual (Chiron Automated Corneal Shaper)	1062 + 182 * (only myopic)	0.4% (partial in 0.6%)	1.8%	0.2%	0.2%		
**Lin and Maloney 1999** [49]	manual (Chiron Automated Corneal Shaper)	1019	2.0%	2.2%		1.1%	1.8%	
**Recep et al., 2000** [101]	manual (Moria micokeratome)	1481	1.42%					
**Clare et al., 2011** [87]	manual (Moria One Use-Plus)	23,997	0.033% **					
**Muñoz et al., 2006** [82]	femtosecond (AMO IntraLase 15 and 30 kHz)	765						1.3%
**Stonecipher et al., 2006** [81]	femtosecond (IntraLase)	5667						1.1%
Sutton and Hodge 2008 [102]	femtosecond (AMO IntraLase 15 and 30 kHz)	1000	0.4%	0.0%	0.0%		0.2%	0.0%
**Haft et al., 2009** [55]	femtosecond (AMO IntraLase 15 and 30 kHz)	4772					0.42%	0.25%
**Clare et al., 2011** [87]	femtosecond (IntraLase FS-60)	57,241	0.003% **					
**de Paula et al., 2012** [78]	femtosecond (AMO IntraLase 60 kHz)	801					12.4%	
**Tomita et al., 2013** [76]	femtosecond (Femto LDV–IntraLase 60 kHz)	818					8.17% –37.5%	

* LASIK enhancement procedures; ** difference statistically significant, and more common in hyperopia. One eye lost 2 lines of BCVA. Abbreviations: DLK—diffuse lamellar keratitis; TLSS—transient light sensitivity syndrome.

### 3.4. Other Considerations

While the cost of using a femtosecond laser for flap creation may only be a small proportion (27–117 Euros per procedure) of the total LASIK cost [103], the cost of acquiring and maintaining the femtosecond laser should still be considered. In some cases, it is possible to use the same platform for LASIK and for femtosecond laser-assisted cataract surgery (FLACS; e.g., Alcon LenSx, Bausch and Lomb Victus, Ziemer Femto LDV Z8). However, some systems dedicated to flap creation are not capable of performing FLACS (e.g., Ziemer Femto LDV Z2/Z4/Z6), and some FLACS systems are unable to create LASIK flaps (e.g., Johnson and Johnson Catalys and the LensAR system) [104].

Another advantage of having a femtosecond laser in the refractive suite is the option to perform small incision lenticule extraction (SMILE). The benefits of SMILE include lower corneal biomechanical impact, less dry eye risk, and less stromal bed exposure [105,106]. Only some femtosecond lasers can perform SMILE procedures; primarily SMILE was developed by Carl Zeiss Meditec and was available only for VisuMax lasers. Other companies have also been working on introducing SMILE into their lasers, and recently Schwind has enabled lenticule extraction in the Atos platform. Moreover, the application of the femtolaser laser could improve the outcomes of many other corneal procedures, such as lamellar keratoplasty, arcuate keratotomy, or finally, those requiring to create corneal pockets (i.e., for intracorneal ring segments implantation, corneal inlays, or stem cell implantation) [104,107,108].

One of the most threatening complications of refractive surgery is corneal ectasia. A high percentage of tissue altered was found to be the most important risk factor for ectasia after LASIK in eyes with normal preoperative corneal topography [109]. Creating a significantly thicker flap than intended would increase the percentage of tissue altered. In a single study, five out of 96 keratoconus suspect eyes (5.2%) developed ectasia after MMK LASIK, while none out of 44 keratoconus suspect eyes in the femtosecond laser group [110]. Nevertheless, a Hansatome MMK was used within the aforementioned study, and this MMK creates relatively thick flaps with a greater variability flap thickness. Moshirfar et al. reported a very low prevalence of ectasia (0.05%; 1/2000 patients) following femtosecond LASIK [111] (Table 3). Another study by Said et al. noted ectasia in 14 out of 6941 eyes (0.2%) following MMK LASIK [112]. In older investigations employing mainly MMKs, the risk for ectasia reached up to 0.9% [110]. Potentially, the improvements in screening methods, as well as in techniques for flap creation, both in femtosecond lasers but also in MMKs, could lead to a decrease in the risk of ectasia in recent years. However, to categorically demonstrate the difference in the prevalence of ectasia following femtosecond and MMK LASIK, a separate meta-analysis should be conducted.

The retreatment rate in older studies was reported as 6.3–10.5% [113,114]. Currently, it is significantly lower. For example, a recent study involving 2581 myopic eyes and using the IntraLase RS laser with a custom ablation profile reported a retreatment rate of 3.2% [115]. In an investigation by Chua et al. the overall retreatment rate was 2.55% (1 335 out of 42 396 eyes); in the early years (1998–2003), it reached 4%, while after 2010, the retreatment rate dropped to <1.2% [16]. Notably, since 2007, the femtosecond laser was employed for flap creation in all surgeries in the study [16]. Pokroy et al. reported a 1.8% overall retreatment rate after MMK LASIK (with a Moria MMK, 90 µm thickness plate) in 9177 eyes; as in other studies, it decreased over time from 2005 to 2012 [22]. Presumably, the low retreatment rate in current studies is not only associated with the use of the femtosecond laser, but with improvements in excimer laser treatment planning and the use of analytic engines, which might take into consideration the possible myopic regression after LASIK.

**Table 3:** Pros and cons of femtosecond LASIK.

Pros	Cons
− Greater predictability of flap thickness creation − Possibility to redo flap creation (also with different depth settings) − Less common complications associated with flap creation (e.g., flap slippage) − A more versatile tool—possibility to employ small-incision lenticule extraction (SMILE) in some platforms or, e.g., to create corneal pockets	− Uncommon complications specific for femtosecond lasers include opaque bubble layer, vertical gas breakthrough, and transient light sensitivity syndrome − Higher rate of diffuse lamellar keratitis − High cost for the laser and its maintenance

## 4. Conclusions

This analysis has shown that the flaps created by femtosecond lasers have higher predictability in terms of flap thickness when compared with flaps created with mechanical microkeratomes. However, newer mechanical microkeratome designs might have similar predictability to femtosecond lasers. Each method has complications—the complications typical for femtosecond lasers (i.e., OBL, TLSS) might be easier to manage than those related to MMKs (e.g., flap displacement). There is no evidence for the unequivocal superiority of one technique over the other.

## Supplementary Materials

The following are available online at www.mdpi.com/article/10.3390/diagnostics11091588/s1.

## Appendix A

**Table A1:** Flap thickness obtained with manual and femtosecond methods for corneal flap creation in clinical LASIK studies.

Study	Number of Eyes	Method of Flap Creation	Intended Thickness [μm]	Method for Flap Thickness Measurement (Device Name)	Measured Central Flap Thickness ±SD [μm]	Timing of Flap Thickness Measurement
Manual microkeratomes						
Kezirian and Stonecipher 2004 [116]	126	Carriazo-Barraquer (Moria)	130	USP (DGH Pachette 50/60 KHz)	153 ± 26	After lifting the flap (total thickness − thickness after flap lift)
Kezirian and Stonecipher 2004 [116]	143	Hansatome (Bausch & Lomb)	180	USP (DGH Pachette 50/60 KHz)	156 ± 29	After lifting the flap (total thickness − thickness after flap lift)
Choudhri et al., 2005 [32]	138	Hansatome (Bausch & Lomb)	160	USP (American Surgical Instruments Corneal Gauge)	124.1 ± 17.4	After lifting the flap (total thickness − thickness after flap lift)
Choudhri et al., 2005 [32]	112	Hansatome (Bausch & Lomb)	180	USP (American Surgical Instruments Corneal Gauge)	142.3 ± 19.6	After lifting the flap (total thickness − thickness after flap lift)
Duffey 2005 [23]	42	LSK-1 (Moria)	100	USP (DGH Pachette)	107.0 ± 14.0	After lifting the flap (total thickness − thickness after flap lift)
Talamo et al., 2006 [35]	100	LSK-1 (Moria)	160	USP (DGH Pachette II)	130.0 ± 19.0	After lifting the flap (total thickness − thickness after flap lift)
Talamo et al., 2006 [35]	135	M2 (Moria)	130	USP (DGH Pachette II)	142.0 ± 24	After lifting the flap (total thickness − thickness after flap lift)
Javaloy et al., 2007 [40]	100	M2 (Moria)	160	confocal microscopy (ASL 165a)	149.1 ± 14.0	1 and 3 months postop
Patel el al., 2007 [26]	21	Hansatome (Bausch & Lomb)	180	confocal microscopy (Nidek ConfoScan 3 or 4)	138.0 ± 22	1 month postop
Huhtala et al., 2007 [34]	300	M2 (Moria)	120	USP (Cilco)	115.4 ± 12.5	After lifting the flap (total thickness − thickness after flap lift)
Alió and Piñero 2008 [6]	22	M2 (Moria)	110	high-frequency USP (Ultralink Artemis)	117.5 ± 7.8	1 month postop
Alió and Piñero 2008 [6]	22	Carriazo-Pendular Microkeratome (Schwind)	110	high-frequency USP (Ultralink Artemis)	118.1 ± 8.3	1 month postop
Hamilton et al., 2008 [30]	32	One Use (Moria)	130	USP (N/A)	117.0 ± 16.0	After lifting the flap (total thickness − thickness after flap lift)
Rosa et al., 2009 [20]	20	Hansatome Zero Compression (Bausch & Lomb)	160	USP (Sonogage Corneo-Gage Plus)	149.1 ± 24.9	After lifting the flap (total thickness − thickness after flap lift); in 1of 4 group 20 min postop
Rosa et al., 2009 [20]	20	Zyoptix XP (Bausch & Lomb)	120	USP (Sonogage Corneo-Gage Plus)	124.7 ± 23.8	After lifting the flap (total thickness − thickness after flap lift); in 1of 4 group 20 min postop
Salomão et al., 2009 [31]	70	Hansatome (Bausch & Lomb)	180	USP (N/A)	131.0 ± 25	After lifting the flap (total thickness − thickness after flap lift)
Ahn et al., 2011 [27]	52	M2 (Moria)	130	OCT (Optovue RTVue FD-OCT)	126.0 ± 19.9 **	2 months postop
Yao et al., 2011 [37]	38	M3 (Moria)	110	OCT (Carl Zeiss Visante)	112.2 ± 5.4	1 week–6 months postop
Zhang et al., 2014 [117]	50	M2 (Moria)	110	USP (DGH 550)	133.0 ± 13.9	After lifting the flap (total thickness − thickness after flap lift)
Zhang et al., 2014 [33]	60	One Use-Plus SBK (Moria)	110	USP (DGH 550)	109.4 ± 6.8	After lifting the flap (total thickness − thickness after flap lift)
Abdelwahab and Elfayoumi 2016 [52]	500	One Use-Plus SBK (Moria)	100	USP (DGH 55 Pachmate)	102.0 ± 6.1	After lifting the flap (total thickness − thickness after flap lift)
Torky et al., 2017 [118]	23	M2 (Moria)	N/A	USP (Tomey SP 100)	104.6 ± 20.1	After lifting the flap (total thickness − thickness after flap lift)
Karabela et al., 2017 [119]	72	M2 (Moria)	120	USP (Nidek Echoscan US-1800)	134.2 ± 19.9	After lifting the flap (total thickness − thickness after flap lift)
Eldaly et al., 2019 [28]	22	M2 (Moria)	100–110	OCT (Heidelberg Engineering Spectralis)	125.8 ± 5.8	1 month postop
Eldaly et al., 2019 [28]	22	M2 (Moria)	130	OCT (Heidelberg Engineering Spectralis)	146.9 ± 13.6	1 month postop
Femtosecond laser						
Kezirian and Stonecipher 2004 [116]	106	IntraLase S3 (Abbott Medical Optics)	130	USP (DGH Pachette 50/60 KHz)	114 ± 14	After lifting the flap (total thickness − thickness after flap lift)
Binder 2004 [120]	34	IntraLase S3 (Abbott Medical Optics)	110	USP (Sonnogage Cornea Scan II 5)	125.0 ± 12.0	After lifting the flap (total thickness − thickness after flap lift)
Binder 2004 [120]	22	IntraLase S3 (Abbott Medical Optics)	120	USP (Sonnogage Cornea Scan II 5)	122.4 ± 11.9	After lifting the flap (total thickness − thickness after flap lift)
Binder 2004 [120]	21	IntraLase S3 (Abbott Medical Optics)	130	USP (Sonnogage Cornea Scan II 5)	128.7 ± 16.6	After lifting the flap (total thickness − thickness after flap lift)
Binder 2004 [120]	26	IntraLase S3 (Abbott Medical Optics)	140	USP (Sonnogage Cornea Scan II 5)	132.5 ± 18.5	After lifting the flap (total thickness − thickness after flap lift)
Talamo et al., 2006 [35]	99	IntraLase FS (Abbott Medical Optics)	110	USP (DGH Pachette II)	119.0 ± 12	After lifting the flap (total thickness − thickness after flap lift)
Javaloy et al., 2007 [40]	100	IntraLase FS (Abbott Medical Optics)	120	confocal microscopy (ASL 165a)	130.1 ± 1.7	1 and 3 months postop
Patel el al., 2007 [26]	21	IntraLase 15- kHz (Abbott Medical Optics)	120	confocal microscopy (Nidek ConfoScan 3 or 4)	143.0 ± 16	1 month postop
Alió and Piñero 2008 [6]	22	IntraLase 30- kHz (Abbott Medical Optics)	110	high-frequency USP (Ultralink Artemis)	116.0 ± 6.2	1 month postop
Hamilton et al., 2008 [30]	32	IntraLase 60- kHz (Abbott Medical Optics)	110–120	USP (N/A)	120.0 ± 13	After lifting the flap (total thickness − thickness after flap lift)
Sutton and Hodge 2008 [102]	838	IntraLase 15- kHz (Abbott Medical Optics)	105	USP (Sonogage Corneo-Gage Plus)	116.8 ± 10.8	After lifting the flap (total thickness − thickness after flap lift)
Sutton and Hodge 2008 [102]	162	IntraLase 30- kHz (Abbott Medical Optics)	115	USP (Sonogage Corneo-Gage Plus)	114.0 ± 9.8	After lifting the flap (total thickness − thickness after flap lift)
Rosa et al., 2009 [20]	20	IntraLase FS 60 kHz (Abbott Medical Optics)	120	USP (Sonogage Corneo-Gage Plus)	115.5 ± 12.5	After lifting the flap (total thickness − thickness after flap lift); in 1of 4 group 20 min postop
Salomão et al., 2009 [31]	113	IntraLase 30- or 60- kHz (Abbott Medical Optics)	100–110	USP (N/A)	131.0 ± 25	After lifting the flap (total thickness − thickness after flap lift)
Ahn et al., 2011 [27]	50	IntraLase	110	OCT (Optovue RTVue FD-OCT)	130.3 ± 13.2 **	2 months postop
Ahn et al., 2011 [27]	40	VisuMax (Carl Zeiss Meditec)	110	OCT (Optovue RTVue FD-OCT)	133.9 ± 13.9 **	2 months postop
Ahn et al., 2011 [27]	64	Femto LDV (Ziemer)	110	OCT (Optovue RTVue FD-OCT)	105.8 ± 8.2 **	2 months postop
Yao et al., 2011 [37]	25	VisuMax (Carl Zeiss Meditec)	100	OCT (Carl Zeiss Visante)	114.2 ± 6.9	1 week–6 months postop
Kim et al., 2011 [36]	19	IntraLase (Abbott Medical Optics)	110	OCT (Carl Zeiss Visante)	115.2 ± 5.0	1 week postop
Kim et al., 2011 [36]	7	IntraLase (Abbott Medical Optics)	120	OCT (Carl Zeiss Visante)	121.9 ± 5.8	1 week postop
Kim et al., 2011 [36]	9	IntraLase (Abbott Medical Optics)	130	OCT (Carl Zeiss Visante)	134.4 ± 5.0	1 week postop
Zhang et al., 2014 [117]	72	FS200 (Alcon)	110	USP (DGH 550)	105.5 ± 5.9	After lifting the flap (total thickness − thickness after flap lift)
Zhang et al., 2014 [33]	60	FS200 (Alcon)	110	USP (DGH 550)	112.7 ± 7.2	After lifting the flap (total thickness − thickness after flap lift)
Zheng et al., 2015 [24]	200	FS200 (Alcon)	110	OCT (OptoVue RTVue FD-OCT)	105.4 ± 3.4	1 week postop
Zheng et al., 2015 [24]	200	VisuMax (Carl Zeiss Meditec)	110	OCT (OptoVue RTVue FD-OCT)	110.8 ± 3.9	1 week postop
Liu et al., 2016 [38]	200	FS200 (Alcon)	110	OCT (OptoVue RTVue FD-OCT)	105.4 ± 4.5	1 week postop
Liu et al., 2016 [38]	200	IntraLase FS60 (Abbott Medical Optics)	110	OCT (OptoVue RTVue FD-OCT)	109.2 ± 11.6	1 week postop
Torky et al., 2017 [118]	26	Visumax FSL (Carl Zeiss Meditec)	100	USP (Tomey SP 100)	100.1 ± 16.1	After lifting the flap (total thickness − thickness after flap lift)
Eldaly et al., 2019 [28]	25	FS200 (Alcon)	100–110	OCT (Heidelberg Engineering Spectralis)	114.6 ± 6.1	1 month postop
Eldaly et al., 2019 [28]	24	FS200 (Alcon)	130	OCT (Heidelberg Engineering Spectralis)	140.8 ± 13.8	1 month postop
Parafita-Fernandez et al., 2020 [39]	44	FS200 (Alcon)	120	OCT (Zeiss Cirrus HD-OCT 5000)	132.2 ± 8.1	1 day–3 months postop

Abbreviations: OCT—optical coherence tomography, SD—standard deviation, USP—ultrasound pachymetry; ** central thickness in horizontal plane.
