# Supplementary material for: Flap Thickness and the Risk of Complications in Mechanical Microkeratome and Femtosecond Laser In Situ Keratomileusis: A Literature Review and Statistical Analysis
Source: Diagnostics (Basel). 2021 Aug 31;11(9):1588. doi: 10.3390/diagnostics11091588 (PMC8468565; doi:10.3390/diagnostics11091588)
Supplement: Supplementary file 1 [file diagnostics-11-01588-s001.zip › diagnostics-1328710-supplementary.pdf]

Supplementary Materials

Search strategy

Literature searches of the PubMed and Web of Science databases were conducted in Jul 15, 2021; the search strategies are as follows. Specific limited update searches were conducted after Jul 15, 2021.

1. PubMed Search (Publication Date 1/1/1900–7/15/2021)

((“corneal flap”[Title]) OR (“femtosecond laser”[Title]) OR (“mechanical microkeratome”[Title])) AND ((“laser in situ keratomileusis”[Title]) OR (“laser-assisted in situ keratomileusis”[Title]) OR (“LASIK”[Title])). 362 references.

2. Scopus (Publication Date 1/1/1900–7/15/2021)

(TITLE(“corneal flap”) OR TITLE(“femtosecond laser”) OR TITLE(“mechanical microkeratome”)) AND (TITLE(“laser in situ keratomileusis”) OR TITLE(“laser-assisted in situ keratomileusis”) OR TITLE(“LASIK”)). 476 references

After removing duplicates 495 publications were analyzed. Finally, 114 articles were considered as relevant.
